# Building to learn: Information technology innovations to enable rapid pragmatic evaluation in a learning health system

**DOI:** 10.1002/lrh2.10420

**Published:** 2024-04-16

**Authors:** Geetanjali Rajamani, Genevieve B. Melton, Deborah L. Pestka, Maya Peters, Iva Ninkovic, Elizabeth Lindemann, Timothy J. Beebe, Nathan Shippee, Bradley Benson, Abraham Jacob, Christopher Tignanelli, Nicholas E. Ingraham, Joseph S. Koopmeiners, Michael G. Usher

**Affiliations:** ^1^ Medical School University of Minnesota Minneapolis Minnesota USA; ^2^ Center for Learning Health Systems Sciences University of Minnesota Minneapolis Minnesota USA; ^3^ Department of Surgery University of Minnesota Minneapolis Minnesota USA; ^4^ Institute for Health Informatics University of Minnesota Minneapolis Minnesota USA; ^5^ Information Technology Fairview Health Services Minneapolis Minnesota USA; ^6^ School of Public Health University of Minnesota Minneapolis Minnesota USA; ^7^ Department of Medicine University of Minnesota Minneapolis Minnesota USA; ^8^ Department of Pediatrics University of Minnesota Minneapolis Minnesota USA; ^9^ Department of Medicine Indiana University Bloomington Indiana USA

**Keywords:** health information technology, learning health systems, pragmatic trials

## Abstract

**Background:**

Learning health systems (LHSs) iteratively generate evidence that can be implemented into practice to improve care and produce generalizable knowledge. Pragmatic clinical trials fit well within LHSs as they combine real‐world data and experiences with a degree of methodological rigor which supports generalizability.

**Objectives:**

We established a pragmatic clinical trial unit (“RapidEval”) to support the development of an LHS. To further advance the field of LHS, we sought to further characterize the role of health information technology (HIT), including innovative solutions and challenges that occur, to improve LHS project delivery.

**Methods:**

During the period from December 2021 to February 2023, eight projects were selected out of 51 applications to the RapidEval program, of which five were implemented, one is currently in pilot testing, and two are in planning. We evaluated pre‐study planning, implementation, analysis, and study closure approaches across all RapidEval initiatives to summarize approaches across studies and identify key innovations and learnings by gathering data from study investigators, quality staff, and IT staff, as well as RapidEval staff and leadership.

**Implementation (Results):**

Implementation approaches spanned a range of HIT capabilities including interruptive alerts, clinical decision support integrated into order systems, patient navigators, embedded micro‐education, targeted outpatient hand‐off documentation, and patient communication. Study approaches include pre‐post with time‐concordant controls (1), randomized stepped‐wedge (1), cluster randomized across providers (1) and location (3), and simple patient level randomization (2).

**Conclusions:**

Study selection, design, deployment, data collection, and analysis required close collaboration between data analysts, informaticists, and the RapidEval team.

## 1 INTRODUCTION

Learning health systems (LHSs) are defined by the Agency for Healthcare Research and Quality (AHRQ) as systems in which “internal data and experience are systematically integrated with external evidence, and that knowledge is put into practice,”^1^ resulting in higher quality care for patients. Pragmatic clinical trials fit well within LHSs as they combine real‐world data and experiences with a degree of methodological rigor which supports generalizability.^2^ However, the intervention being tested within a pragmatic trial must also be tailored to a given LHS setting to maximize success. This includes obtaining input and buy‐in from clinical leaders, health information technology (HIT), and other shared service operations while also incorporating prospective control groups that allow for hypothesis testing.

Leveraging the electronic health record (EHR) to support recruitment, treatment assignment, and intervention deployment in pragmatic trials is gaining popularity.^3^, ^4^ Multiple examples of pragmatic trials have used a range of technology tools, for example, best practice alerts [BPAs], and workflows in the EHR, demonstrating the potential to make a large impact on quality, cost, as well as both patient and care team experience.^3^, ^4^

While pragmatic trials are a promising method for conducting LHS research, they also present unique challenges. For example, a study by Richesson et al. surveyed 20 teams who used the EHR for pragmatic trials to understand the challenges they faced and solutions they developed.^5^ The authors found that 55% of projects had difficulties with IT staff turnover, and integration of data from heterogeneous systems. About 50% of teams reported difficulties with utilizing the EHR for intervention delivery. In response to these challenges, Richesson et al. suggested certain prerequisites that healthcare systems and EHRs should have before conducting pragmatic trials. These included ensuring adequate IT staff, standardizing data collection and validation, and building flexible, scalable processes that can be adapted to support additional trials in a standard and replicable fashion.^5^

Initially deployed in late 2021, the Rapid Prospective Evaluation Unit (RapidEval) at the University of Minnesota's Center for Learning Health Systems Sciences (CLHSS) was designed to drive rapid, iterative learning that melds pragmatic trial design with mixed methods to support innovation in healthcare.^6^ To further advance the field of LHS, we conducted a detailed characterization of the role of HIT, including innovative solutions and challenges that occur, in improving LHS project delivery. Specifically, this report not only details what challenges arose in our LHS when implementing pragmatic trials in the EHR, but also what IT innovations were created and implemented to address these challenges.

## 2 METHODS

**FIGURE 1:**
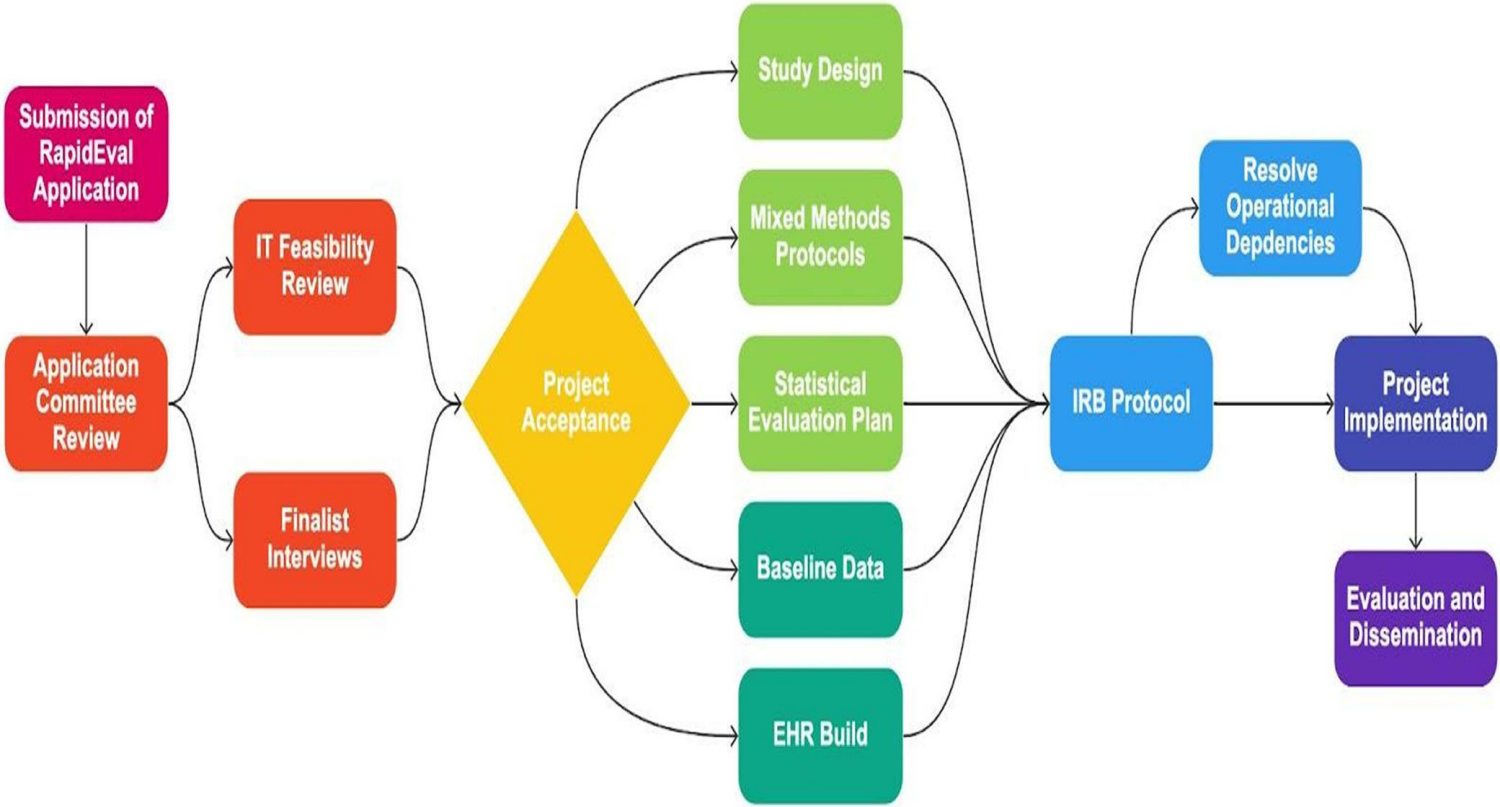
RapidEval application and implementation process.

This study was conducted by the RapidEval Unit at the University of Minnesota's CLHSS in Minneapolis, Minnesota between December 2021 and February 2023. During this time period, there were four “calls for proposals” for RapidEval projects. Calls for proposals were advertised widely among University and CLHSS staff and consisted of an application and an interview. Thus far, there have been 51 applications, of which eight have been selected based on feasibility of implementation within 2–3 months, potential for positive impact on healthcare delivery or health equity, alignment with health system priorities, low‐risk nature of interventions, and impact on the science of healthcare delivery. Five projects are ongoing, one is in pilot testing, and two are in the planning phase. During the first year of RapidEval, we developed a unique process to evaluate, plan, and implement RapidEval projects (Figure 1).

**FIGURE 2:**
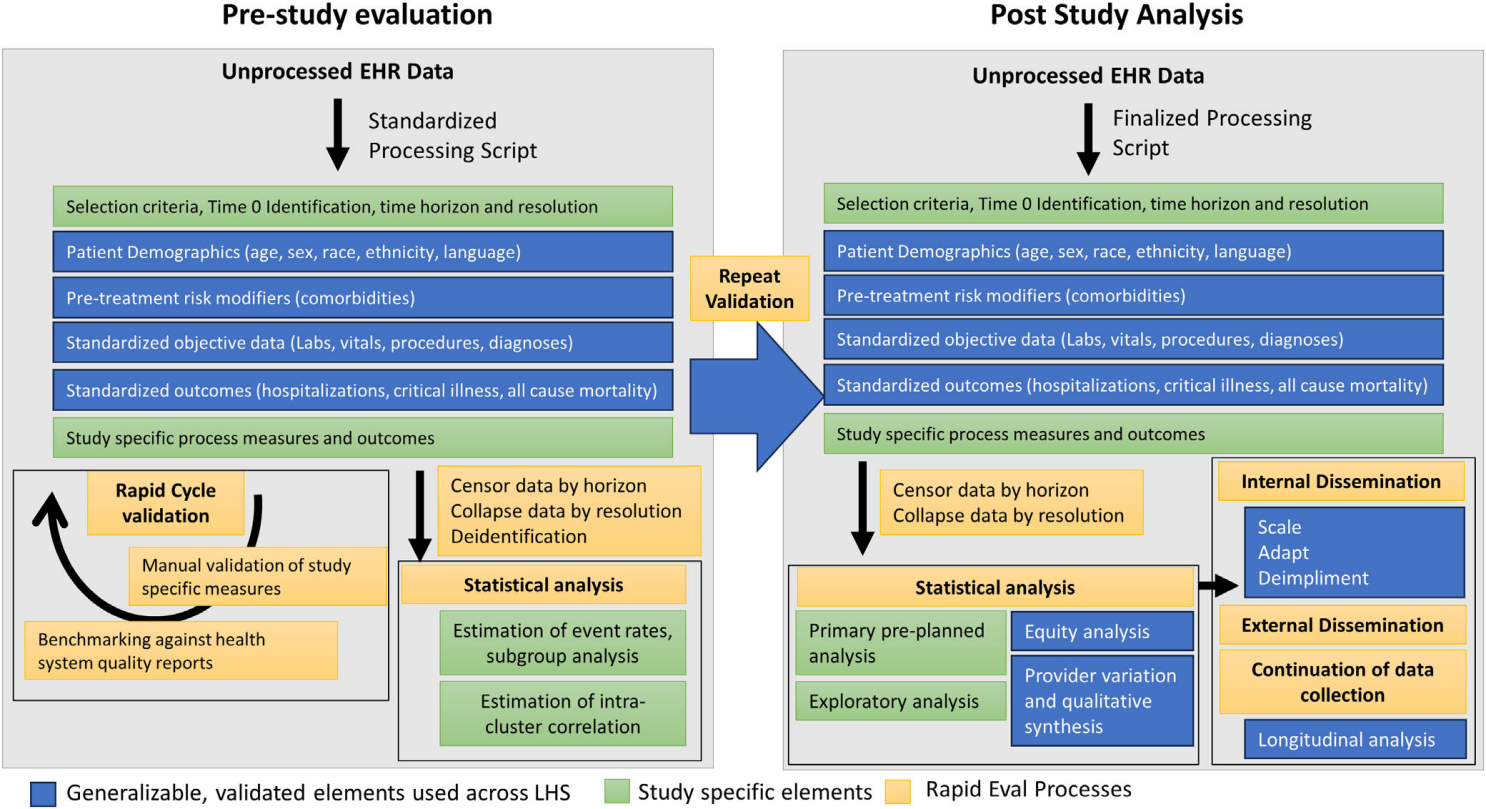
RapidEval pre‐study evaluation and post‐study analysis processes.

In order to support rapid cycle planning and evaluation, we needed to design infrastructure that standardized data collection, validation, and analysis (Figure 2). The general approach was to leverage predefined logic to process raw EHR data into useful categories including demographics, comorbidities, structured data, and outcomes which is continually validated for accuracy by the LHS. For each study, inclusion criteria and definition of a Time‐0 were created to approximate when a patient may be recruited in collaboration with clinical leadership and stakeholders. For example, this could be the start of a hospitalization or outpatient encounter, or when a specific test was ordered. Then, a time horizon (length of time to collect data) and resolution (frequency of sampling and summarizing objective data) were decided. This could be augmented by study specific process measures and outcomes. As such, manual validation then focuses primarily on study‐specific measures (inclusion criteria, process measures). Data were censored, dropping data that fell outside of the study horizon. Data were then collapsed into average, minimum, maximum, and trajectory across predefined time windows (resolution). Finally, the datasets were deidentified using the safe‐harbor approach,^7^ and used to support baseline analysis including estimating baseline event rates, intra‐class correlation, and parameters needed to support study design. All data processing was directed by health system and LHS analysts within the health system's computing environment.

This standardized approach to data collection and validation, which facilitated timely data analysis by maintaining a consistent data structure across projects, eased analysis by statisticians by maintaining data in the same format across widely variable projects. Moreover, it supports subsequent structured dissemination which includes recommendations to the health system, such as to broadly scale the intervention, adapt the intervention, or de‐implement. In addition to public dissemination, this approach supports continued longitudinal data collection to facilitate additional exploratory analysis outside of the scope of RapidEval, and internal development of quality measures within the health system.

Implementation approaches spanned a range of EHR capabilities including: interruptive alerts (e.g., asking patients about medication affordability), clinical decision support integrated both in the order systems and patient navigators for both inpatient and outpatient encounters (e.g., suggesting providers adhere to cardiac monitoring guidelines), targeted outpatient hand‐off documentation (e.g., targeting hand‐off to include methods to reduce surgical site infections), and patient messaging (e.g., in an app tailored to enable responsible patient opioid use). Study approaches include pre‐post with time‐concordant controls (1), non‐randomized staggered intervention (1), randomized stepped‐wedge (1), cluster randomized design (3), and patient level randomization (2). Pre‐study planning required a collaborative approach between data analysts, informaticists, the RapidEval team, and clinical stakeholders to support rapid cycle planning and development of interventions which could be deployed in a manner which maximized statistical rigor (Table 1). Some challenges associated with these study approaches and interventions included: identifying variations in provider behaviors to better provide clinical decision support, embedding educational materials into the EHR, designing a communication platform to best reach patients after surgery, and more (see Table 1).

**TABLE 1:** Overview of RapidEval projects and associated challenges.

Short project title	Baseline cohort definition	Study design	Intervention	Unique challenges	Anticipated outcome
Reduction in telemetry overuse and provider variation	Any patient admitted to the hospital	Pre‐post with concordant controls	Clinical decision support and best practice alert (BPA) that encourages adherence to American Heart Association (AHA) guidelines for cardiac monitoring for indication and duration	Algorithmically capturing provider variation on a day‐to‐day basis	A reduction in the overall utilization of cardiac monitoring and an understanding of why providers order telemetry in excess of guidelines
				Identifying variation in provider behavior	
				Understanding impact to workflow of clinical support staff	
Embedded clinical prediction of chemotoxicity among older adults with cancer	Patient older than 65 with an oncology appointment and diagnosis of a solid tumor	Cluster randomized at the provider level	Cancer and Aging Research Group (CARG) chemotoxicity prediction tool embedded into the EHR	Gated patient navigator elements by provider based on randomization	A reduction in admissions related to chemotoxicity and increase in palliative care referrals, and an understanding of the role of chemotoxicity prediction in patients newly diagnosed with cancer
				Handling missing data while automating incorporation of structured content	
EHR embedded micro‐education to improve antibiotic timing in sepsis	Patients admitted to the hospital meeting systemic inflammatory response syndrome (SIRS) criteria, lactate >2.0, and antibiotics initiated	Randomized stepped‐wedge	A short educational video directed at nurses to provide rationale for sepsis goals and educate on current system efforts	Embedding educational materials into the EHR and tracking engagement	A reduction in the time from antibiotic order to administration. An understanding of barriers and facilitators to rapid administration of antibiotics
				Assessing educational responses across all nurses within the system	
Decreasing opioid addiction and diversion using behavioral economics applied through a digital engagement solution	Opioid naive patients with recent elective surgery and postoperative opioid prescribing	Sequential adaptive trial with patient level randomization	A messaging service that provides standardized educational information on opioids tailored to patients depending on their input and response	Designing and implementing a communication platform to assess and respond to postoperative pain	A reduction in number of patients on opiates after 14 days of surgery. An improved understanding of postoperative pain and pain control within the context of an app‐based communication tool
				Embedding randomization into access for patients who meet criteria	
				Pre‐planned randomization strategy to support subgroup stratification and adaptation for future versions	
Improving equity of medical therapy management (MTM) referrals	Patients admitted to the hospital with 10 or more medications and a recent readmission	Pilot pre‐post study followed by patient level randomized trial	Asking patients about medication affordability at the point of care and automating referral to pharmacy services for patients at high risk for readmission	Embedding assessment of medication affordability at the point of care	An improvement in care coordination and reduction in readmissions among patients with difficulty affording their medications.
				Adapting automation to respond to system level changes in the MTM referral process	
Assessing the impact of a telestroke program on stroke center utilization and quality of care	Patients who present to the emergency department with acute ischemic stroke	Non‐randomized staggered intervention	System‐wide adoption of telestroke consultation for all community hospitals	Improving efficiency of telestroke scheduling given limited number of platforms	A reduction in the number of inter‐hospital transfers for stroke without a reduction in quality. An understanding of the barriers and facilitators of high‐quality stroke care through tele‐stroke at community hospitals
				Assessing impact when many patients transfer out of system	
				Harmonizing and merging naturally collected EHR data with manually collected registry data	
Standardizing communication to reduce surgical site infection (SSI) risk	Patients admitted with a surgery high risk for surgical site infection	Cluster randomized at the hospital level	Standardizing hand‐off and debrief procedures to include SSI reduction bundle	Streamlining the hand‐off procedure to reduce burden and improve adherence	Improve adherence to the SSI bundle and improvement in SSI rates. An understanding of barriers and facilitators to SSI bundle adherence
				Tailoring the intervention to address process variations at different hospitals	
Improving pre‐exposure prophylaxis prescribing (PrEP) to high‐risk patients in the outpatient setting	Patients identified as high risk by sexually transmitted infection (STI) testing, positivity, and social history	Cluster randomized at the clinic level	Standardized order set to streamline STI testing, and embed a CDS to inform patients and simplify PrEP prescribing	Building a new process that overcomes provider discomfort without increasing burden	Improving the rate of PrEP prescribing among patients with high risk for HIV Better capturing of HIV risk in the EHR
				Improving collection of sexual orientation and gender identity data	

For each project, a work group was generated including health system leaders, study team leads, CLHSS leadership, IT leadership, and critical stakeholders. Decisions regarding underlying study design, including randomization strategy, and specifics of the intervention were made by this group, which leaned heavily on health system IT leadership and staff. The overall direction of RapidEval, and large decisions including study selection, funding and support, study closure, and dissemination were overseen by a steering committee comprised of CLHSS and RapidEval leadership.

## 3 IMPLEMENTATION/RESULTS

We found that integrating health system IT stakeholders was critical for all phases of a project, including proposal evaluation, planning, implementation, and analysis. Throughout this process, IT stakeholders highlighted the importance of operational buy‐in (Table 2, “Pre‐Study Planning”). Specifically, they mentioned that while other health systems have separate groups for IT operations versus research, M Health Fairview (the healthcare system affiliated with University of Minnesota) has one joint group working with the Center for Learning Health Systems Sciences, thus establishing a culture of research within IT. Furthermore, obtaining operational buy‐in was necessary for seeing the RapidEval projects through. This included emphasizing the benefits of research in the IT space (e.g., added flexibility to projects), leveraging congruencies between existing work in the system and research efforts, and creating relationships with IT staff. Stakeholders also highlighted the randomization of interventions (Table 2, “Implementation”), a difficult challenge to overcome given the technical limitations of the EHR, as a solution that would not have been created if not for RapidEval. Note that the “stakeholders” mentioned include project managers and IT personnel of various RapidEval projects who volunteered time for an open‐ended interview with study authors.

**TABLE 2:** Challenges and information technology innovations in implementing RapidEval projects at each study phase.

Study phase	Challenges	Innovations
Pre‐study planning	Defining baseline cohort definitions and measures from raw EHR data	Adaptive process that collapses raw EHR data into a standardized research database
	Building a research database to support final study design, randomization approach, and power	Rapid cycle validation of definitions through manual chart review while logic is being defined
		Share de‐identified baseline data with study statisticians
	Obtaining operational buy‐in to incorporate research objectives alongside IT team goals; mismatch between resource availability and team readiness	Integrate research objectives into regular operational intake processes; establish a culture of research for shared understanding; emphasize the flexibility that research can introduce into IT projects; leverage existing support of education for new initiatives
Implementation	Designing EHR based intervention	Dedicated IT project management to assist with resourcing and navigating the organization
	Implementing with controls to reduce bias	Embedding location‐based limitations or random number generators to assign clinical pathways
	Prospectively updating research database incorporating study goals	Dedicated analyst time to identify new measures that capture adherence and outcomes
Data collection and analysis	Assessing implementation	Leverage existing frameworks to evaluate implementation using mixed methods for all projects
	Finalizing statistical analysis in practice embedded studies	Creation of a dedicated research environment within the health system, for study statisticians to access directly
Study closure	Ending the project to free‐up resources efficiently	Creation of a framework to de‐implement, adapt, or scale depending on findings
	Ensuring learning continues	Allowing data to continue to accrue, and share it with established clinical research infrastructure
	Disseminating findings to operational stakeholders	Yet to be addressed—potentially via creation of committees to disseminate results and revisit and de‐implement as appropriate

IT personnel were instrumental in identifying barriers, specific implementation strategies, and data acquisition approaches. For example, in the reduction of telemetry study, the original plan was to conduct as a cluster randomized trial; however, this would have required disentangling over 200 order bundles. This approach was not deemed feasible, and an alternative approach was identified. Another example involved the chemotoxicity study. An initial primary measure was the portion of patients with a dose reduction after initiation of chemotherapy. Dedicated IT staff highlighted the difficulty of determining this measure given the complexity of chemotherapy data within the EHR.

Other unique innovations detailed in Table 2 were used to overcome encountered challenges included: creating a dedicated research environment for statisticians to access in order to embed analyses into projects; creating a de‐implementation framework in order to end projects efficiently and free‐up resources; and allowing data to accrue and share it so that learning continues.

This work highlights multiple important impacts of a practice‐embedded pragmatic trial unit within a learning health system. This includes development of local expertise to not only develop a broad range of process improvement efforts but methods to improve the evaluation of innovations. The focus on structured data collection allowed the creation of multiple validated measures of clinical performance, health equity, and practice variation to support both research and operations. Finally, due to tight integration with the health system IT resources, RapidEval projects have led to the creation of a multitude of structured interventions with broad reach and impact on patient care.

## 4 CONCLUSION

This case report details important IT innovations which enable rapid pragmatic trials leveraging the EHR. There were three critical components for success. First, direct collaboration with clinical informatics stakeholders is necessary in all phases of study selection, planning, and implementation. This helps ensure that selected projects are actually feasible and ensures smooth implementation. Second, standardized data collection with focused validation facilitates rigorous study planning and rapid analysis. We found that maintaining consistent data structures across projects eased statistical analysis and furthermore eases dissemination of data and results to the health system and even public. Finally, fostering local expertise in implementation and structured deployment that utilizes randomization, when possible, eases future study planning (an outcome which we hope to see as RapidEval continues in the years to come). IT infrastructure to support pragmatic trials directly supports innovations in patient care with wide reach and rigorous assessment to guide future planning, while simultaneously enabling the production of generalizable knowledge, meeting the aims of a learning health system.

## CONFLICT OF INTEREST STATEMENT

The authors have no relevant conflicts of interest to report.
